# The Prediction of Outcomes in Patients Admitted With Traumatic Brain Injury Using the Rotterdam Score

**DOI:** 10.7759/cureus.29787

**Published:** 2022-09-30

**Authors:** Farrukh Javeed, Lal Rehman, Mehar Masroor, Maham Khan

**Affiliations:** 1 Neurosurgery, Jinnah Postgraduate Medical Centre, Karachi, PAK; 2 Neurosurgery, Aga Khan University Hospital, Karachi, PAK; 3 Radiation Oncology, Aga Khan University Hospital, Karachi, PAK

**Keywords:** prognosis, glasgow outcome score, glasgow coma scale, rotterdam score, traumatic brain injury

## Abstract

Objective: The objective was to use the Rotterdam score, which is based on a CT scan, to assess the outcomes of traumatic brain injury patients.

Material and Methods: This research, which included 319 head trauma patients, was carried out at the neurosurgery department of a tertiary care hospital between June 2019 and December 2020. The Rotterdam score was calculated for each patient on the basis of the first CT scan after the head injury. The Glasgow Outcome Score was used to assess the results three months following the injury.

Results: In our research, there were 270 male patients (84.6%) and 49 female patients (15.4%). The mean age was 37.4 ± 15.4 years and road traffic accidents were observed in 275 people (86.2%). Severe traumatic brain injury (TBI) was seen in 123 patients (38.6%). The most common Rotterdam score was 2 in 86 (27.0%) patients, while it was score 3 in 72 (22.6%), score 4 in 59 (18.5%), score 5 in 41 (12.9%), score 1 in 31 (9.7%) and score 6 in 29 (9.1%). The mortality rate was 33.5% in our patients and good recovery was seen in 150 (47.0%) patients.

Conclusion: The Rotterdam score is a useful tool to evaluate and predict outcomes in head trauma patients.

## Introduction

Traumatic brain injury (TBI) is brain damage induced by an external mechanical impact that results in impaired brain function [1]. It is a severe, global health issue responsible for 70% of mortality in trauma patients, with nearly 200 cases per 100,000 people reported annually [2]. TBI is much more prevalent in men than in women and is a major cause of morbidity and mortality among young individuals [3]. In Pakistan, the most prevalent cause of TBI is road traffic accident, followed by fall, gunshot injury, and assault, though rates of head trauma due to fall have been increasing [4-5]. The Glasgow Coma Scale (GCS), a widely used grading system for determining a patient's level of consciousness after TBI, classifies injury levels as mild, moderate, or severe [6]. However, the inter-rater reliability of the GCS is poor because of the subjective variations, especially when the verbal response cannot be evaluated (e.g., in individuals undergoing tracheal intubation) [7]. The GCS also cannot be utilized to predict prognosis because it does not provide the true functional status of a patient [8].

TBIs are classified as primary or secondary. Primary injury occurs immediately after the initial trauma and may include intracranial hemorrhages and skull fractures that can damage the brain or lead to infection. Primary injuries can be diffuse or localized [9]. Secondary injury develops gradually over days or even weeks and results from subsequent alterations in the microenvironment and cellular mechanisms. To prevent secondary injury, it is critical to diagnose and treat primary injury promptly.

The gold standard imaging modality for assessing and diagnosing patients with TBI is CT scanning, which aids in both the diagnosis and management of potential intracranial injuries that require neurosurgical interventions [10]. In 1991, the Marshall scale became the first CT-based approach for evaluating the prognosis of patients with TBI by assessing the presence of midline shifts, mass lesions, and basal cisterns [10]. In 2005, Maas et al. designed the Rotterdam score system [11], which further assesses epidural lesions, intraventricular blood, and subarachnoid hemorrhage [11]. Specifically, the basal cistern is graded as 0 (normal), 1 (compressed), or 2 (absent); the midline shift is graded as 0 (no shift or shift < 5mm) or 1 (shift > 5mm); the epidural mass lesion is graded as 0 (absent) or 1 (present); and intraventricular blood or subarachnoid hemorrhage is graded as 0 (absent) or 1 (present). With these additional assessments, the Rotterdam score performs better than the Marshall scale at predicting disease outcomes in patients with TBI [11]. The goal of this research was to evaluate the efficacy of the Rotterdam score in outcome prediction for patients with TBI.

## Materials and methods

This study included 319 consecutive patients aged 16 years or above hospitalized in our department with brain injury from June 2019 to December 2020. We excluded patients with a previous history of head trauma or head surgery and those with polytrauma. The following continuous and categorical variables were analyzed for mean, range, and frequencies using IBM SPSS Statistics for Windows, Version 22.0 (Released 2013; IBM Corp., Armonk, New York, United States): age, gender, mode of injury, time since injury, duration of hospital stay, GCS on admission, Rotterdam score on admission, type of management, and outcome based on Glasgow Outcome Score at three months after the head injury. The chi-square test was applied to calculate the relationship between variables and outcome and a p-value of less than 0.05 was taken as significant.

## Results

Our research included 319 individuals with a mean age of 37.4 years, 270 (84.6%) of whom were male and 49 (15.4%) who were female. The most common mode of injury was road traffic accident in 275 (86.2%) cases, followed by fall in 40 (12.5%) cases. Table 1 summarizes the demographic data and characteristics of the patients.

**Table 1:** Demographic data of patients with traumatic brain injury (N=319)

Variable			Number of Patients		Percentage	
Gender	Male	270		84.6		
	Female	49		15.4		
Cause of injury	Road traffic accident	275		86.2		
	Fall	40		12.5		
	Firearm	2		0.6		
	Assault	1		0.3		
	Others	1		0.3		
Time since injury	<1 hour	38		11.9		
	1–3 hours	264		82.8		
	4–6 hours	12		3.8		
	>6 hours	5		1.6		
Glasgow Coma Scale on admission	Mild head injury	99		31.0		
	Moderate head injury	97		30.4		
	Severe head injury	123		38.6		
Management	Conservative	235		73.7		
	Surgery	84		26.3		
Length of stay	<24 hours	111		34.8		
	1–3 days	89		27.9		
	4–7 days	70		21.9		
	>7 days	49		15.4		
Mortality	Yes	107		33.5		
	No	212		66.5		
Glasgow Outcome Scale	1	107		33.5		
	2	10		3.1		
	3	17		5.3		
	4	35		11.0		
	5	150		47.0		

Most (264, 82.8%) patients reached the hospital within 1-3 hours of injury. At the time of admission, GCS scores for head injury were severe in 123 (38.6%) patients, moderate in 97 (30.4%), and mild in 99 (31.0%). Rotterdam score on admission was 1 in 31 (9.7%) patients, 2 in 86 (27.0%) patients, 3 in 72 (22.6%) patients, 4 in 59 (18.5%) patients, 5 in 41 (12.9%) patients, and 6 in 29 (9.1%) patients, as shown in Table 2.

**Table 2:** Rotterdam score and rate of mortality in patients with traumatic brain injury (N=319)

Rotterdam score	Number of patients (%)	Mortality n (%)
1	31 (9.7)	1 (3.2)
2	87 (27.0)	15 (17.2)
3	72 (22.6)	18 (25)
4	59 (18.5)	23 (38.9)
5	41 (12.9)	27 (65.8)
6	29 (9.1)	23 (79.3)

Surgical treatment was performed in 84 (26.3%) patients, and 235 (73.7%) were managed conservatively. The duration of hospital stay was less than 24 hours for 111 (34.8%) patients, 1-3 days for 89 (27.9%) patients, 4-7 days for 70 (21.9%) patients, and more than seven days for only 49 (15.4%) patients. The mean hospital stay in our study was six days. The Glasgow Outcome Score was 1 (death) in 107 (33.5%) patients, 2 (persistent vegetative state) in 10 (3.1%) patients, 3 (severe disability) in 17 (5.3%) patients, 4 (moderate disability) in 35 (11.0%) patients, and 5 (complete recovery) in 150 (47.0%) patients. The relationship between variables and outcome was calculated; age, GCS on admission, and Rotterdam score on admission showed a positive trend (p-value < 0.05) while gender, mode of injury, and time since injury didn’t show any positive relationship (p-value > 0.05).

## Discussion

TBI is a substantial burden on healthcare systems worldwide, with significant mortality and morbidity. Increasing rates of head trauma highlight the need for accurate diagnostic and predicting tools. Assessments of head injury can occur at the site of the event, at accident and emergency departments, and throughout the patient’s hospital stay. Various TBI scoring systems using different clinical and radiological parameters are available to aid in TBI outcome prediction.

In our studied patients with traumatic brain injury, young male patients aged 30-40 years were the most common, which aligns with published studies showing that young males are the most prevalent patients with brain injuries [12-14]. In our study, more than two-thirds of TBIs were due to road traffic accident injuries, which mirrors results from other studies [12,13,15]. We found that most (302, 94.7%) patients arrived at the hospital within three hours of head injury, compared to 12 (3.8%) who arrived within four to six hours and only 5 (1.6%) who arrived more than six hours after the head injury. The duration between injury and arrival at the hospital has an important role in patient outcomes because it is observed that prolonging duration not only delays the treatment of a head injury patient but also can lead to the progression of intracranial hematoma and mass effect. 

Initial assessment at our hospital revealed that about one-third of the patients acquired severe head trauma, compared to more than half of patients in a 2018 study using CT scores to assess TBI [15]. This difference was mainly because the other study only included patients with moderate and severe head injuries while our study also included mild head injury patients. In our study, the most common Rotterdam score was 2 or 3, which was the score for about half of our studied patients, whereas a score of 6 was assigned to only one out of ten patients. In a study by Huang et al. [12], nearly two-thirds of their patients received a Rotterdam score of 5 or 6; in a study by Majdan et al. [13], two-thirds received a Rotterdam score of 2 or 3. Only 84 (26.3%) in our study underwent surgical treatment, compared to 235 (73.7%) who were managed conservatively. It demonstrates that the majority of patients merely require hospitalization for observation and that they do not require any form of surgical intervention whatsoever. Only a minority of patients end up having surgical procedures done to treat head injuries. About one-third stayed in the hospital for less than 24 hours or longer than three days (34.8% versus 37.3%, respectively). One study from Taiwan showed that the mean hospital stay for TBI patients was 34 days [12], compared to six days in our study. This disparity is primarily the result of two factors: first, the patients in our study were more likely to have suffered mild or moderate brain injuries, and second, our institute has a policy that allows patients who are vitally and neurologically stable and do not require surgical intervention to be discharged early and sent home.

Complete recovery with full functional status was observed in about half of our patients. One-third expired during the study period. The remaining one-fifth had varying degrees of functional disability. Other studies also have shown high incidences of unfavorable outcomes among patients with TBI, including a mortality rate of one in three in patients with a mean follow-up of over a year [12], bad outcomes in 46% of patients at 30 days of follow-up [16], and mortality rates of 41% and 22%, respectively [13,15].

We observed a statistically significant positive association between patient age and the Glasgow Outcome Scale (p-value of 0.00), indicating that the mortality rate after TBI increases with age. Other studies have shown conflicting results of either no relation between age and the Glasgow Outcome Scale or a significant relation [14-15]. Although most patients in our research were male, there was no statistically significant association between gender and outcome (p-value > 0.05). There was no significant link between the mode of injury and the Glasgow Outcome Scale (p-value > 0.05).

In our study, time since injury had no impact on outcomes (p-value > 0.05). The relation between GCS on admission and the Glasgow Outcome Scale was significant, however (p-value = 0.00). Severe head injuries were associated with higher rates of poor prognosis, similar to another study showing a significant relation between GCS and Rotterdam score [14]. The relation between the Rotterdam score on admission and the Glasgow Outcome Scale was also significant (p-value < 0.05), as shown in table 3, with the highest mortality (23 patients, 79.3%) associated with a Rotterdam score of 6.

**Table 3:** Rotterdam score and patient outcome in patients with traumatic brain injury (N=319)

		Glasgow Outcome Score					Total	p-value
		1	2	3	4	5		
Rotterdam score on admission	1	1	1	0	6	23	31	0.00
	2	16	2	9	10	50	87	
	3	18	2	2	10	40	72	
	4	23	2	6	5	23	59	
	5	26	1	0	4	10	41	
	6	23	2	0	0	4	29	
Total		107	10	17	35	150	319	

This finding somewhat differs from the two other studies reporting high mortality rates (67% and 58%, respectively) for patients with a Rotterdam score of 6 [12,17]. Additional studies also have shown that the Rotterdam score can significantly predict mortality in head trauma patients [14-15]. Patients who had a poor outcome grade had a longer hospital stay (p-value < 0.05), but the type of management had no observable effect on their outcomes.

The Rotterdam scoring system has significant limitations, one of which being the fact that even little changes in how the score is predicted by different observers can have a significant impact on prognosis [17]. Nevertheless, the Rotterdam score outperforms other CT-based scoring systems with its own distinct CT characteristics, making it a superior prognostic model [15,17]. Despite its weaknesses, it is a simple and practical option with good prognostic value in patients with TBI [12,18-20]. This study is limited by its reliance on data from a single tertiary care hospital. Future research should aim to include additional centers and increase the sample size.

## Conclusions

A continued and extensive medical innovation and research to look for an optimal strategy to predict head trauma injuries is still being sought. By assessing various injury aspects (e.g., epidural lesions, intraventricular blood, subarachnoid hemorrhage, basal cistern, midline shift, and epidural mass), the Rotterdam score outperforms other available tools for predicting disease outcomes in these patients. Our study showed that the Rotterdam score is a useful predictor of outcomes in head trauma, particularly mortality, even compared to numerous other predictive scoring systems.
